# Prevalence of human papillomavirus types and variants and p16^INK4a^ expression in head and neck squamous cells carcinomas in São Paulo, Brazil

**DOI:** 10.1186/s13027-016-0067-8

**Published:** 2016-05-04

**Authors:** Julio C. Betiol, Laura Sichero, Henrique O. de Olival Costa, Leandro L. de Matos, Maria A. Andreoli, Silvaneide Ferreira, Sheila F. Faraj, Evandro S. de Mello, João S. Sobrinho, Lenine G. Brandão, Claudio R. Cernea, Marco A. Kulcsar, Fabio R. Pinto, Antonio J. Gonçalves, Marcelo B. Menezes, Leonardo Silva, Lia M. Rossi, Rafaella A. Lima Nunes, Lara Termini, Luisa L. Villa

**Affiliations:** Molecular Biology Laboratory, Center of Translational Research in Oncology, Instituto do Câncer do Estado de São Paulo, Hospital das Clínicas da Faculdade de Medicina da Universidade de São Paulo, São Paulo, Brazil (ICESP), São Paulo, Brazil; Department of Otolaringology, Santa Casa de Sao Paulo, School of Medicine (FCMSCSP), São Paulo, Brazil; Department of Head and Neck Surgery, School of Medicine, University of São Paulo, São Paulo, Brazil; HPV Institute, Santa Casa de Sao Paulo, School of Medicine (FCMSCSP), São Paulo, Brazil; Department of Pathology, Cancer Institute of São Paulo (ICESP), São Paulo, Brazil; Department of Surgery, Santa Casa de Sao Paulo, School of Medicine (FCMSCSP), São Paulo, Brazil; Department of Radiology and Oncology, School of Medicine, University of São Paulo, São Paulo, Brazil; Center of Translational Oncology - ICESP, Av. Dr. Arnaldo, 251, 8 andar, 01246-000, Cerqueira César, São Paulo, SP Brazil

**Keywords:** Human papillomavirus, Head and neck cancer, Molecular variants, p16^INK4a^, Prevalence

## Abstract

**Background:**

Human papillomavirus (HPV) prevalence in head and neck squamous cell carcinomas (HNSCC) diverges geographically. The reliability of using p16^INK4a^ expression as a marker of viral infection is controversial in HNSCC. We evaluated HPV types and HPV-16 variants prevalence, and p16^INK4a^ expression in HNSCC specimens provided by two different Institutions in São Paulo.

**Methods:**

HPV DNA from formalin-fixed specimens was accessed by Inno-LiPA, HPV-16 variants by PCR-sequencing, and p16^INK4a^ protein levels by immunohistochemistry.

**Results:**

Overall, HPV DNA was detected among 19.4 % of the specimens (36/186). Viral prevalence was higher in the oral cavity (25.0 %, 23/92) then in other anatomical sites (oropharynx 14,3 %, larynx 13.7 %) when samples from both Institutions were analyzed together. HPV prevalence was also higher in the oral cavity when samples from both Institutions were analyzed separately. HPV-16 was the most prevalent type identified in 69.5 % of the HPV positive smaples and specimens were assigned into Asian-American (57.2 %) or European (42.8 %) phylogenetic branches. High expression of p16^INK4a^ was more common among HPV positive tumors.

**Conclusion:**

Our results support a role for HPV-16 in a subset of HNSCC.

## Background

Worldwide head and neck squamous cell carcinoma (HNSCC) is the sixth most common type of cancer with an estimated annual incidence of approximately 600,000 cases [1]. HNSCC comprises squamous cell carcinomas (SCC) of the oral cavity, oropharynx, larynx and hypopharynx. In Brazil, head and neck cancer is the fourth and seventh most incident tumor among man and women, respectively [2].

Although alcohol and tobacco consumption is the leading predisposing risk factor for HNSCC development [3], there are epidemiological evidences that human papillomavirus (HPV) infection further plays an etiological role. In fact, since 2009, the International Agency for Research on Cancer recognizes HPV-16 as an independent causal agent of oropharyngeal SCC, while the carcinogenic effect upon the oral cavity and the larynx is still a matter of debate [4]. HPV prevalence in HNSCC ranges from 10 % to 90 % dependent of the geographical region and the anatomical site of the tumor [5]. HPV-related and unrelated HNSCC diverge considerably at the genetic, molecular, epidemiological, and clinical level [6]. HPV-positive HNSCC are more common among young adults and non-smokers, and these individuals respond better to treatment and have better survival than their HPV-negative counterparts [7].

HPV-16 intratype nucleotide heterogeneity has been extensively studied [8]. Based on differences in identity of less than 2 % within the *L1* gene, HPV-16 molecular variants are clustered in five branches of phylogenetic and geographical relatedness: European (E), Asian-American (AA), Asian (As), African-1 (Af-1) and African-2 (Af-2) [9, 10]. The association of HPV-16 non-European variants with increased risk of developing cervical intraepithelial neoplasia (CIN) was observed in several studies in Brazil and the United States [11–13].

The detection of HPV DNA in HNSCC is not a sufficient proof for causal viral association. High-risk HPV E7 inactivates the retinoblastoma protein, thus promoting p16^INK4^ overexpression. In the cervix, p16^INK4a^ upregulation is being used as an indirect measure of oncogenically active HPV infection [14]. Although HPV DNA detection associated to high levels of p16^INK4a^ has also been used as surrogate marker of biologically relevant viral infections in the oropharynx [15], for other head and neck subsites data is still inconsistent. It is crucial to precisely identify HPV-driven HNSCC in each population in order to improve clinical management of this disease, and to evaluate the potential benefits of the prophylactic vaccines available. Our aim was to analyze the prevalence of HPV types and HPV-16 variants in HNSCC from different anatomical sites of individuals treated at two Institutions in the city of São Paulo, and further evaluate p16^INK4a^ protein levels in these samples.

## Methods

### Clinical samples

Inclusion criteria consisted of histological confirmation of HNSCC, information about the year of diagnosis and no treatment prior to surgical procedure. Formalin-fixed Paraffin Embedded (FFPE) tumor specimens from 96 HNSCC patients diagnosed between 1991 and 2010 were retrieved from the pathology archives of the Santa Casa de Sao Paulo, School of Medicine (FCMSCSP). Further, 109 FFPE HNSCC specimens from patients treated at the Cancer Institute of São Paulo (ICESP) from 2009 to 2012 were included in this study. Samples were selected from the pathology services of both institutions following a chronological sequence, availability and quality of specimen (for molecular analyses). All sections were reviewed by a pathologist of each participant Institution. Based on anatomical subsite localization of the tumor, specimens were categorized as oral cavity (oral tongue, gum, mouth, floor of mouth, lips), oropharynx (base of tongue, tonsil, oropharynx), and larynx, according to The International Classification of Diseases criteria of the World Health Organization. The ethics committees of both institutions approved all study procedures.

### DNA Extraction and HPV detection

About ten 6 μm paraffin sections were obtained; the first and last sections were submitted to histological analysis, while the remaining was submitted to DNA isolation. After removing paraffin with xylene, tissues were digested with proteinase K 1 mg/mL-SDS 0.1 % for 24 h. DNA was obtained by organic extraction: after addition of Phenol/Chloroform/Isoamyl Alcohol at 25:24:1 followed by vigorous homogenization and centrifugation, DNA precipation was conducted with ethanol 100 %. The DNA pellet was dried and was dissolved in 100 μL of TE. DNA concentration was determined with a Nanodrop 2000 spectrophotometer (Thermo Scientific, MA, USA). Samples were diluted to 50 ng/uL and 2 μl were used in each PCR. DNA quality was assessed by amplification of a 110 bp fragment of the human β-globin gene using PCO3/PCO4 primers followed by analysis in 8 % acrylamide gel electrophoresis [16]. The Inno-LiPA HPV Genotyping kit (Innogenetics, Gent, Belgium) was used for HPV DNA detection and genotyping as described [17]. This kit identifies 28 different HPV types by reverse blot hybridization: 6, 11, 16, 18, 26, 31, 33, 35, 39, 40, 43, 44, 45, 51, 52, 53, 54, 56, 58, 59, 66, 68, 69, 70, 71, 73, 74, 82.

### HPV-16 variant characterization

A 193 bp segment of the LCR (nt 7744–33) (Forward CTAACCTAATTGCATATTTGG; Reverse ACGCCCTTAGTTTTATACATG) and/or a 108 bp fragment of the *E6* gene (nt 266–374) (Forward AGAGATGGGAATCCATATGC; Reverse: GCTGTTCTAATGTTGTTCCATAC) were amplified using AmpliTaq Gold polymerase (Perkin-Elmer, CA, USA). PCR products were cloned with TOPO TA Cloning® kit for Sequencing (Invitrogen, CA, USA). Sequencing was conducted in a PRISM® 3100 Genetic Analyser (AB Applied Biosystems, CA, USA) using the BigDye Terminator v3.1 Cycle Sequencing kit (AB Applied Biosystems, CA, USA). Sequences alignment was achieved using Bioedit 7.2.5, and HPV-16 variants were defined as previously described [8, 10].

### P16^INK4a^ expression

Immunohistochemistry for p16 ^INK4a^ was performed using the CINtec® P16^INK4a^ Histology Detection kit (Roche Diagnostics GmbH, Mannheim, Germany) in a Ventana Benchmark GX equipment (Ventana Medical Systems, Arizona, USA) according to the manufacturer's instructions. Staining of the samples from both institutions (FCMSCSP and ICESP) were analyzed by the same pathologist at ICESP (S.F.F). Expression of p16^INK4a^ was scored as high (≥75 % of cells stained), low (<75 % of cells stained) or negative (no staining). Diverse studies use this scoring system for defining p16^INK4a^ as positive in oropharyngeal SCC staining [18].

### Statistical analysis

Qualitative data were expressed as absolute numbers and relative rates with 95 % confidence interval (95 % CI), and quantitative data as means, medians and range. The Chi-square test was used to compare phenomena between qualitative variables. For all analysis the probability of a making an α or type I error was equal to or less than 5 % (*P* ≤ 0.05) and SPSS® version 17.0 (SPSS® Inc; Illinois, USA) was used.

## Results

All samples that were negative for human β-globin gene amplification were excluded from the study (*n* = 17); remaining 94 patients from each institution (FCMSCSP and ICESP). The median age of patients from FCMSCSP was 58 years, ranging from 39 to 87 years, and most individuals were male (86 %). Among specimens from ICESP, median age was 61.5 years with a range of 43 to 91 years (Table 1).

**Table 1:** Age and gender of HNSCC individuals from ICESP and FCMSCSP

	Gender	Age (years)			
		*n*	Mean	Median	Range
*FCMSCSP*	Overall	94	59.9	58.0	39-87
	Female	13	64.5	63.0	45-87
	Male^a^	81	59.1	57.5	39-85
*ICESP*	Overall	94	63.4	61.5	43-91
	Female	28	68.6	68.0	43-91
	Male	66	61.2	59.0	44-86

^a^For 5 men enrolled at FCMSCSP no age data available. FCMSCSP-Santa Casa de Sao Paulo, School of Medicine; ICESP-Cancer Institute of São Paulo

Concerning samples obtained from FCMSCSP, most tumors were from the larynx (*n* = 59, 62.8 %), followed by the oral cavity (*n* = 22, 23.4 %) and the oropharynx (*n* = 11, 11.7 %). For two samples (2.1 %) the anatomic site could not be defined due to the extension of the tumor, and both specimens were also excluded from analysis. Most cases from ICESP were derived from the oral cavity (*n* = 70, 74.5 %), followed by the oropharynx (*n* = 17, 18.0 %) and the larynx (*n* = 7, 7.5 %).

**Fig. 1:**
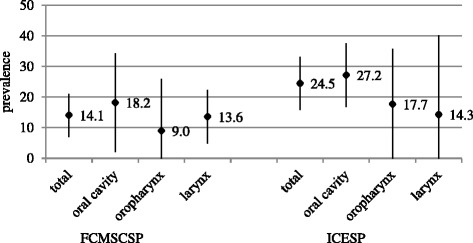
Overall HPV DNA prevalence by anatomical subsite and sample origin. Prevalence (%) and 95 % CI are indicated. FCMSCSP-Santa Casa de Sao Paulo, School of Medicine; ICESP-Cancer Institute of São Paulo

The presence of HPV DNA was investigated in all 186 cases of HNSCC (92 from FCMSCSP and 94 from ICESP). When the two cohorts were analyzed together, overall HPV DNA prevalence was 19.4 % (36/186); HPV prevalence was higher in the oral cavity (25.0 %, 23/92), and similar viral DNA rates were observed in the oropharynx (14.3 %, 4/28) and the larynx (13.7 %, 9/66). HPV prevalence was also the highest in the oral cavity when samples from both institutions were analyzed separately: 18.2 % and 27.2 % of the samples from FCMSCSP and ICESP were PCR positive, respectively (Fig. 1). While among samples from FCMSCSP viral prevalence was higher in larynx as compared to the oropharynx, the opposite was observed in ICESP specimens. Nevertheless, none of the differences observed are statistically significant (*P* > 0.05). For both series of cases, HPV-16 was the most commonly detected viral type, identified in 69.5 % (25/36) of the HPV positive specimens (Table 2). Co-infections by HPV-16 and HPV-18 were detected in two oral cavity samples from ICESP.

**Table 2:** HPV type specific prevalence by anatomical subsite and sample origin

	HPV neg	HPV pos	HPV6	HPV11	HPV16	HPV18	HPV35	HPV52	HPV58	HPV74
*FCMSCSP*										
oral cavity	18	4		2	2					
oropharynx	10	1					1			
larynx	51	8^a^			6			1		
*ICESP*										
oral cavity^b^	51	19	1		14	4			1	1
oropharynx	14	3			3					
larynx	6	1				1				

^a^One of the samples was HPV positive but viral type was not discriminated using Inno-Lipa

^b^Two cases of multiple infection with HPV-16 and HPV-18

FCMSCSP-Santa Casa de Sao Paulo, School of Medicine; ICESP-Cancer Institute of São Paulo. Neg-negative. Pos-positive

We initially thought to characterize HPV-16 molecular variants based in the nucleotide sequence pattern of a fragment of the *E6* gene. However, we were unable to amplify this viral segment in six samples. For this reason, we further performed a PCR using primers able to amplify a segment of the HPV-16 LCR. Even though, we had no success in the amplification of four specimens. Overall, we detected European and Asian-American HPV-16 variants in 12 (57.2 %) and 9 (42.8 %) of the specimens, respectively (Table 3).

**Table 3:** HPV-16 molecular variants by anatomical subsite and sample origin

	HNSCC subsite	HPV-16	AA/NA1	E	Not typed
		(*n*)	*n* (%)	*n* (%)	*n* (%)
*FCMSCSP*	Total	8	1 (12.5)	5 (62.5)	2 (25.0)
	Oral cavity	2	0 (0)	2 (100.0)	0 (0)
	Oropharynx	0	0 (0)	0 (0)	0 (0)
	Larynx	6	1 (16.7)	3 (50.0)	2 (33.3)
*ICESP*	Total	17	8 (47.1)	7 (41.2)	2 (11.7)
	Oral cavity	14	5 (35.7)	7 (50.0)	2 (14.3)
	Oropharynx	3	3 (100.0)	0 (0)	0 (0)
	Larynx	0	0 (0)	0 (0)	0 (0)

FCMSCSP-Santa Casa de Sao Paulo, School of Medicine; ICESP-Cancer Institute of São Paulo. AA/NA1-Asian-American/North American, E-European

It was possible to evaluate p16^INK4a^ expression by immunohistochemistry in 94 and 87 specimens from FCMSCSP and ICESP, respectively. Table 4 shows the association of HPV positivity and p16^INK4a^ expression in the different HNSCC tumor sites of samples obtained from both institutions. We observed that independently of the anatomical site of the tumor, p16^INK4a^ high expression (>75 % of cells stained) was more common among HPV positive specimens.

**Table 4:** p16^INK4a^ levels by immunohistochemistry among HPV positive and negative HNSCC

	HPV DNA positive			HPV DNA negative		
	p16 neg	p16 low	p16 high	p16 neg	p16 low	p16 high
	*n* (%)	*n* (%)	*n* (%)	*n* (%)	*n* (%)	*n* (%)
*FCMSCSP*						
total	14 (70.0)	3 (15.0)	3 (15.0)	49 (66.2)	22 (29.7)	3 (4.1)
oral cavity	4 (100.0)	0 (0)	0 (0)	9 (56.3)	7 (43.7)	0 (0)
oropharynx	0 (0)	0 (0)	1 (100.0)	5 (55.6)	3 (33.3)	1 (11.1)
larynx	5 (62.5)	1 (12.5)	2 (25.0)	35 (71.4)	12 (24.5)	2 (4.1)
*ICESP*						
total	14 (66.7)	2 (9.5)	5 (23.8)	60 (91.0)	5 (7.5)	1 (1.5)
oral cavity	13 (76.4)	2 (11.8)	2 (11.8)	42 (89.3)	4 (8.5)	1 (2.2)
oropharynx	1 (33.3)	0 (0)	2 (66.7)	12 (92.3)	1 (7.7)	0 (0)
larynx	0 (0)	0 (0)	1 (100.0)	6 (100.0)	0 (0)	0 (0)

FCMSCSP-Santa Casa de Sao Paulo, School of Medicine; ICESP-Cancer Institute of São Paulo

## Discussion

Since the early 1980s morphological and immunohistochemical evidence points towards the involvement of HPV in oral SCC [19]. Furthermore, risk factors for HNSCC are remarkably comparable to those of cervical cancer, comprising multiple sexual partners, younger age at first sexual intercourse and oral sex practice [20, 21]. Actually, over the last decade it became clear that HPV-16 is etiologically linked to a defined subset of HNSCC, mainly in the oropharynx, and more specifically in the tonsils [22]. An etiologic link between HPV and non-oropharyngeal tumors is less firmly established [23, 24].

Reported detection rates of HPV DNA in HNSCC worldwide vary considerably, ranging from 0 % to 100 % in the oropharynx [25]. Nevertheless, HPV is recognized by now as the major cause of oropharyngeal cancer in developed countries, and HPV-16 detection is associated to the rapid rise in the incidence of this neoplasia over the last decade particularly in the USA, Sweden, and Australia, where it causes more than 50 % of cases [26, 27]. Among the HNSCC samples analyzed in our study, overall HPV prevalence was 19.4 %, being highest in the oral cavity as compared to the other anatomical sites. Our results are consistent with another studies conducted in São Paulo and Rio de Janeiro in which HPV prevalence observed was 19.2 % (22/114) and 15.5 % (11/71) in oral SCC, respectively [28, 29], but is otherwise much higher than described in another study involving 132 oral tumor samples from four countries in Latin America including Brazil (HPV prevalence was 0.0 %) [30]. When restricting the comparison solely to laryngeal SCC, we detected HPV DNA in ~14.0 % of the samples from both institutions, similar to previously reported in a study that gathered samples from 4 Brazilian cities [31]. We have also found higher HPV DNA prevalence in oropharyngeal cancers as previously reported on tumors from Brazil [30, 31]. Interestingly, we found differences in oropharyngeal HPV prevalence between the two groups of samples analyzed by us (9.0 % in FCMSCSP and 17.7 % in ICESP). Nevertheless, for all anatomical sites our study has detected lower HPV DNA prevalence when compared to studies conducted in the USA and Europe [5, 26, 32]. Divergence in HPV DNA rates observed could be attributed not only to differences in the methodologies used for HPV detection in the different studies, but also to the characteristics of the individuals under study, including sexual practices, economic and social status. Unfortunately, the lack of sociodemographic and sexual behavior information of the individuals evaluated by us hampers any additional analysis.

Consistent with all studies conducted worldwide HPV-16 was the most prevalent viral type detected independently of the HNSCC anatomical subsite, and was identified in 69.5 % (25/36) of the HPV positive tumors. We and others have reported that in the uterine cervix non-European variants are linked to a higher oncogenic potential [33, 34], and to increased risk of invasive cancer as compared to European isolates [11, 12]. Concerning HPV-16 intratypic analysis in HNSCC, there is still limited data available. Gillison [22] examined HPV-16 sequence variability in 52 HNSCC in the USA and detected a predominance of European variants (75 %), while Asiatic (17 %), North-American (4 %) and African (4 %) variants were less represented. Furthermore, as previously reported for the uterine cervix, in studies conducted in Europe, >90 % of the HPV-16 variants detected in HNSCC belongs to the European branch [35, 36], with the exception of a study from Italy in which about 20 % of the HPV-16 positive cases consisted of African variants [37]. To our knowledge this is the first report concerning description of HPV-16 variability in head and neck tumor specimens in Brazil. Among the HNSCC samples that we were able to characterize the HPV-16 variant, we observe overall slight predominance of European variants followed by Asian-American isolates, comparable to our report on normal cervical samples [11]. In this way it seems that variant distribution reported in HNSCC merely reflects the overall frequency of circulating variants in the populations studied and correlates with the intrinsic admixture level of each population similar to previously reported for the cervical region [38]. It is noteworthy, that all HPV-16 oropharyngeal SCC samples analyzed by us comprise Asian-American variants. Still, the very limited number of samples analyzed precludes further conclusions. Accordingly, natural history studies of HPV in head and neck subsites are crucial to better evaluate the clinical relevance of HPV-16 heterogeneity on viral infection persistence and HNSCC development, predominantly in oropharyngeal SCC where it plays a casual role.

Only the detection of HPV DNA in HNSCC biopsies is not enough evidence of tumor causation and other parameters should be analyzed to prove the pathogenic role of viral infection. Even though overexpression of p16^INK4a^ may also derive from non-viral alterations [39], it has been proven efficient as a surrogate marker of HPV activity in cervical samples [40]. The use of this marker to clinically detect an oncogenically active HPV infection in oropharyngeal SCC has also been described [41]; however, in oral SCC, this correlation is still controversial [42]. We observed that p16^INK4a^ high expression (>75 % cells stained) was more commonly observed in HPV positive tumors, especially among oropharyngeal samples for which 75 % of HPV positive samples overexpressed this protein.

## Conclusions

In summary, we observe that in our population overall HPV prevalence is lower than reported in developed countries, even though HPV-16 was the most prevalent viral type as observed in all studies conducted worldwide. HPV-16 variant distribution detected in our samples seemed to replicate data of the uterine cervix in our population. However, the role of HPV-16 intratypic variability in HNSCC development deserves further analysis. Nevertheless, the fact that HPV16 accounted for almost all HPV positive HNSCC samples indicates that developed prophylactic vaccines for cervical cancer could also be relevant for HNSCC prevention in our population.

## Abbreviations

AA: Asian-American
Af-1: African-1
Af-2: African-2
As: Asian
CIN: cervical intraepithelial neoplasia
E: European
FCMSCSP: Santa Casa de Sao Paulo School of Medicine
FFPE: formalin-fixed paraffin embedded
HNSCC: head and neck squamous cell carcinoma
HPV: human papillomavirus
ICESP: cancer institute of São Paulo
SCC: squamous cell carcinomas
